# Molecular diagnosis of SARS-CoV-2 in seminal fluid

**DOI:** 10.1007/s40618-021-01580-x

**Published:** 2021-04-30

**Authors:** D. Paoli, F. Pallotti, G. Nigro, L. Mazzuti, M. N. Hirsch, M. B. Valli, S. Colangelo, C. M. Mastroianni, G. Antonelli, A. Lenzi, O. Turriziani, F. Lombardo

**Affiliations:** 1 Laboratory of Seminology-Sperm Bank “Loredana Gandini”, Department of Experimental Medicine, “Sapienza” University of Rome, Viale del Policlinico 155, 00161 Rome, Italy; 2 Laboratory of Virology, Department of Molecular Medicine, “Sapienza” University of Rome, Rome, Italy; 3 National Institute for Infectious Diseases, INMI (Istituto Nazionale Per Le Malattie Infettive), “Lazzaro Spallanzani” IRCCS, Rome, Italy; 4 Department of Public Health and Infectious Diseases, “Sapienza” University of Rome, Rome, Italy

**Keywords:** SARS-CoV-2, COVID-19, RT-PCR, Seminal fluid

## Abstract

**Purpose:**

Due to relevant repercussions on reproductive medicine, we aimed to evaluate feasibility of RT-PCR as a detection method of SARS-CoV-2 RNA in seminal fluid.

**Methods:**

A qualitative determination of the RT-PCR assays in semen was performed through different approaches: (1) efficiency of RNA extraction from sperm and seminal plasma was determined using PRM1 and PRM2 mRNA and a heterologous system as control; (2) samples obtained by diluting viral preparation from a SARS-CoV-2 panel (virus cultured in Vero E6 cell lines) were tested; (3) viral presence in different fractions of seminal fluid (whole sample, seminal plasma and post-centrifugation pellet) was evaluated. Semen samples from mild and recovered COVID-19 subjects were collected by patients referring to the Infectious Disease Department of the Policlinico Umberto I Hospital - “Sapienza” University of Rome. Control subjects were recruited at the Laboratory of Seminology-Sperm Bank “Loredana Gandini'' of the same hospital.

**Results:**

The control panel using viral preparations diluted in saline and seminal fluid showed the capability to detect viral RNA presence with *C*_t_ values depending on the initial viral concentration. All tested semen samples were negative for SARS-CoV-2, regardless of the nasopharyngeal swab result or seminal fluid fraction.

**Conclusion:**

These preliminary data show that RT-PCR for SARS-CoV-2 RNA testing appears to be a feasible method for the molecular diagnosis of SARS-CoV-2 in seminal fluid, supported by results of the control panel. The ability to detect SARS-CoV-2 in semen is extremely important for reproductive medicine, especially in assisted reproductive technology and sperm cryopreservation.

## Introduction

Coronaviruses are a family of positive-sense single-stranded RNA viruses that cause infections in birds and mammals as well as humans, inducing respiratory, hepatic, neurological and gastrointestinal diseases [1]. The novel coronavirus SARS-CoV-2 causes pneumonia, a severe acute respiratory disease (COVID-19). Structurally, SARS-CoV-2 is composed of several proteins: nucleocapsid (N), spike (S), membrane (M) and envelope (E). The spike protein is particularly important, as it enables the virus to enter and infect host cells and determines viral pathogenesis, host tropism, and disease [2].

The use of accurate molecular tests has enabled the presence and development of this virus to be monitored. The gold standard for the diagnosis of SARS-CoV-2 infection is qualitative reverse transcription-polymerase chain reaction (qRT-PCR) testing of nasopharyngeal swabs [2, 3]. The usual SARS-CoV-2 gene targets are E, S, N1, N2, and RpRd. The RT-PCR cycle threshold (*C*_t_) value is an indicator of the number of viral copies, with lower *C*_t_ values corresponding to higher viral copy numbers. A *C*_t_ less than 40 is interpreted as positive for SARS-CoV-2 RNA [4]. However, it must be pointed out that *C*_t_ values are not standardized to enable quantification of the viral concentration.

Recently, some authors observed that the N2 gene may be prone to false positive results. Particularly high *C*_t_ values (> 40) have been detected in nasopharyngeal swabs using N2 as the RT-PCR target, suggesting either “very low” viral load or "false positive" results. Careful interpretation of the clinical relevance of this “very low” test result is currently needed [4]. Although respiratory samples are the reference specimens, the virus has been found in numerous human samples, including urine, feces, cerebrospinal fluid, lacrimal fluid and blood [5]. In several studies, bronchoalveolar lavage fluid (BLF) (93%), sputum (72%) pharyngeal swabs (32%), feces (29%), and blood (1%) samples have tested positive. None of the urine samples tested were positive [4, 6]. According to some authors, these different viral loads could be attributed to the sample type or timing, the stage of the disease and/or where the specimen was taken from, all factors that play an important role in RT-qPCR results [7].

Negative test results do not necessarily rule out infection. False negatives can occur in preanalytical steps (poor specimen collection, inappropriate sampling), analytical steps (PCR inhibition, target mutation or low viral load in the sample), and postanalytical steps (transcription error) [3, 8]. In contrast, false positives may arise due to two problems associated with RT-qPCR: contamination and determination of the limit at which it may be affirmed that a sample with a low viral load is in fact positive [9]. Notably, a positive molecular test indicates only the detection of viral RNA and may be unrelated to the presence of infectious virus [8].

In addition to methodological aspects, it should be stressed that a different SARS-CoV-2 level may be the result of different tissue expression of the receptors with which the virus interacts—angiotensin-converting enzyme 2 (ACE2) and transmembrane protease serine 2 (TMPRSS2)—suggesting possible routes of infection other than respiratory droplets. For this reason, research efforts have to date focused on various objectives, including the study of the routes of viral transmission and the research and validation of diagnostic methods.

The impact of SARS-CoV-2 on male reproduction has not yet been established. An important aspect for reproductive medicine is whether or not this virus is found in seminal fluid. While a number of recent literature studies have investigated the presence of SARS-CoV-2 in semen, only one reported positive results, in four acute and two recovering COVID-19 patients (19%) [10]. However, this study may have several major methodological limitations [11].

The identification of SARS-CoV-2 in different clinical samples using RT-PCR is not yet well established. For this reason, the aim of our study was to verify RT-PCR in semen samples, to establish if SARS-CoV-2 is truly found in semen and if this can be used for diagnosis.

## Materials and methods

### Patients

Patients from the Infectious Disease Department of the Policlinico Umberto I Hospital—“Sapienza” University of Rome were asked to provide a semen sample for viral RNA determination. They comprised:

- Mild COVID-19 patients with a recent nasopharyngeal swab positive for SARS-CoV-2 (pt #1 and pt #2).
- Recovered COVID-19 patients with a recent nasopharyngeal swab negative for SARS-CoV-2 **(**pt #3 and pt #4).

Controls were recruited from healthy men attending the Laboratory of Seminology—Sperm Bank “Loredana Gandini'' who were performing semen analysis as a part of pre-conceptional screening and had had a negative nasopharyngeal swab for SARS-CoV-2. All patients provided their written informed consent before any study procedures were carried out. This study was approved by our institution’s Ethics Committee (Ref. 5971, protocol 0646/2020).

### Semen analysis

Semen samples were collected by masturbation into a sterile plastic container. Given the patients’ medical condition, days of abstinence were not taken into consideration. All samples were allowed to liquefy at 37 °C for 60 min and were then assessed according to World Health Organization guidelines (2010). The following variables were assessed: ejaculate volume (ml), sperm concentration (*n* × 10^6^/ml), total sperm number (*n* × 10^6^/ejaculate), progressive motility (%) and morphology (% abnormal forms). A sperm viability test was carried out to differentiate cell death from immotility by staining with eosin Y 0.5% in saline solution.

### Processing of semen samples

**Fig. 1:**
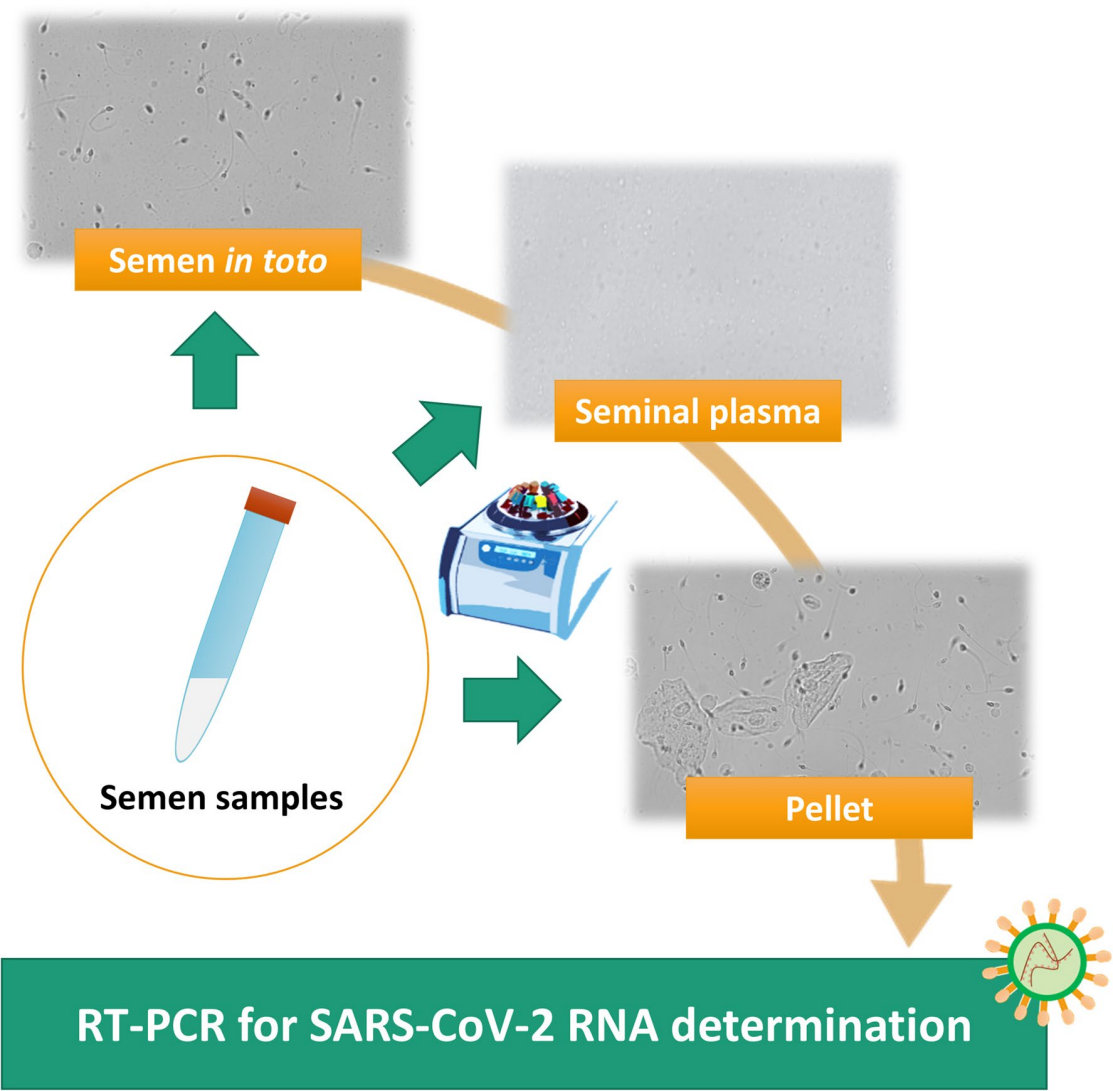
Schematic representation of semen samples processing

To evaluate the possible presence of SARS-CoV-2 in different fractions of seminal fluid, each sample was processed as follows (Fig. 1):

- 140 µl of whole seminal fluid was used to extract Viral RNA.
- An aliquot of seminal fluid was centrifuged at 3000 rpm for 10 min to separate seminal plasma from spermatozoa and other cellular elements. The pellet containing spermatozoa, leukocytes and epithelial cells was diluted with 0.5% saline (cell suspension).
- 140 µl of seminal plasma and 140 µl of cell suspension were used to extract viral RNA.

### Detection of SARS-CoV-2 RNA in semen

Viral RNA from 140 µl of whole seminal fluid, and seminal plasma was extracted using QIAamp viral RNA kit (Qiagen) according to the manufacturer's instructions. Six µl of a heterologous amplification system (Internal Control-RealStar SARS-CoV2 RT PCR, Altona Diagnostics) was used as control for extraction procedure and RT-PCR inhibition. Total RNA extraction from cell suspension was performed using Norgen total RNA purification kit (Norgen Biotek Corporation) according to the manufacturer's instructions. Ten µl of extracted RNA was reverse-transcribed and simultaneously amplified using a real-time RT-PCR system (RealStar SARS-CoV2 RT PCR, Altona Diagnostics) targeting E and S viral genes. PRM1 and PRM2 mRNA, sperm-specific nuclear proteins, was used as the control for sperm RNA extraction, using RT-PCR (TaqMan™ Gene Expression Assay, Applied Biosystems).

### Control panel

**Fig. 2:**
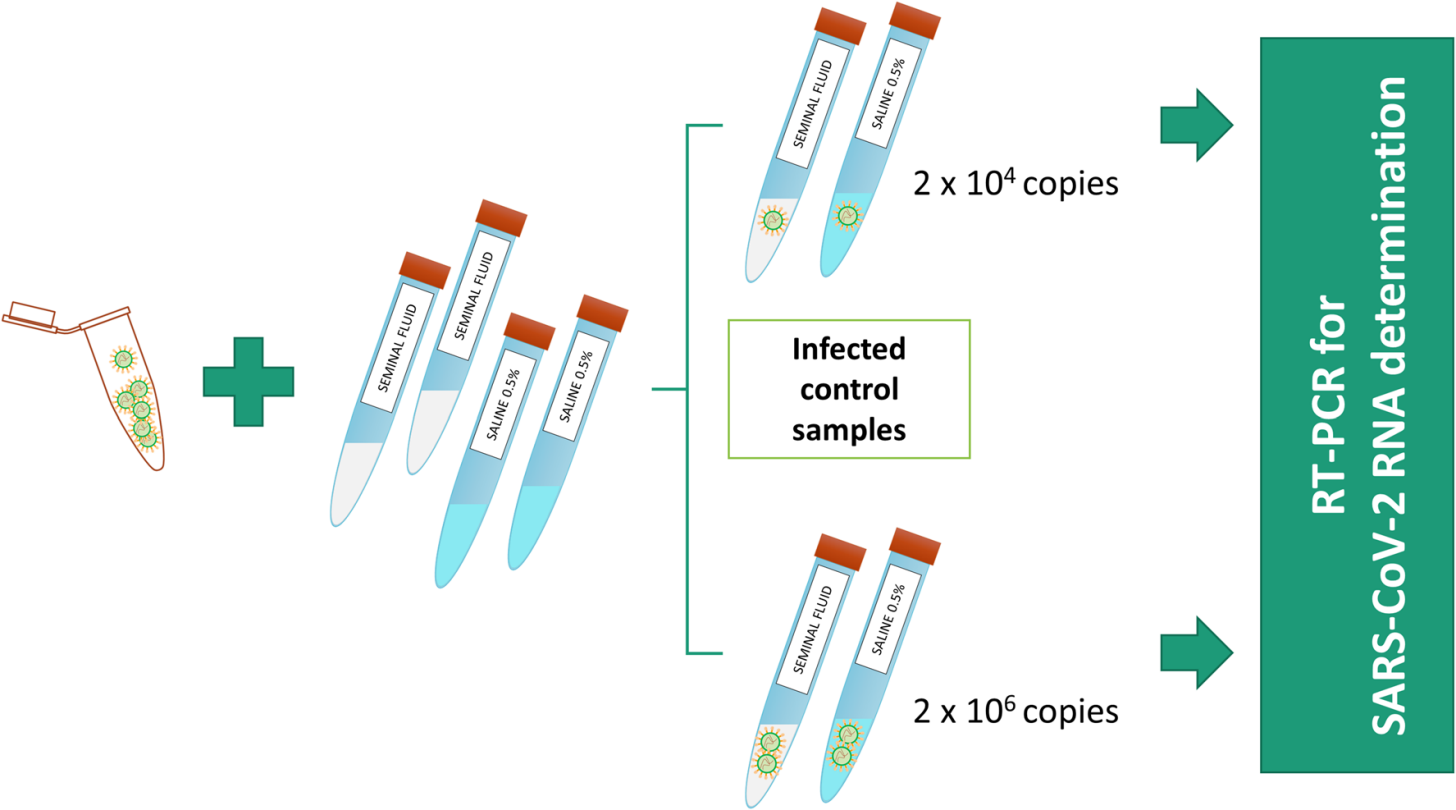
Preparations of control panel samples diluted in seminal fluid and in 0.5% saline solution

To assess whether viral RNA extraction was affected by the use of whole seminal fluid, a known titer of SARS-CoV-2 virus was added to semen from a healthy donor. The panel was prepared with serial dilutions of a SARS-CoV-2 isolate (named 2019-nCoV/Italy-INMI1) [12]. The virus was collected by nasopharyngeal swab and cultured in Vero E6 cell lines grown in MEM containing 2% FBS. The dilutions ranged from 4 × 10^2^ to 4 × 10^6^ viral RNA copies/ml, corresponding to 0.1 and 1000 TCID50 (50% tissue culture infective dose)/ml. SARS-CoV-2 RNA was amplified by qRT-PCR and quantified based on a standard curve prepared through serial dilutions of EURM-019 single-stranded RNA (ssRNA) fragments of SARS-CoV-2 including different target genes (https://crm.jrc.ec.europa.eu/p/EURM-019). Viral titers were determined by limiting dilution assay on Vero E6 cells and infectivity was expressed as TCID50/ml, calculated according to the Reed and Muench method. A blank containing only cell culture medium was included in the panel. Two known titer viral preparations from the panel were diluted 1:2 in seminal fluid and in 0.5% saline solution (Fig. 2). The final concentrations of the tested samples were 2 × 10^4^ and 2 × 10^6^ copies/ml.

## Results

**Fig. 3:**
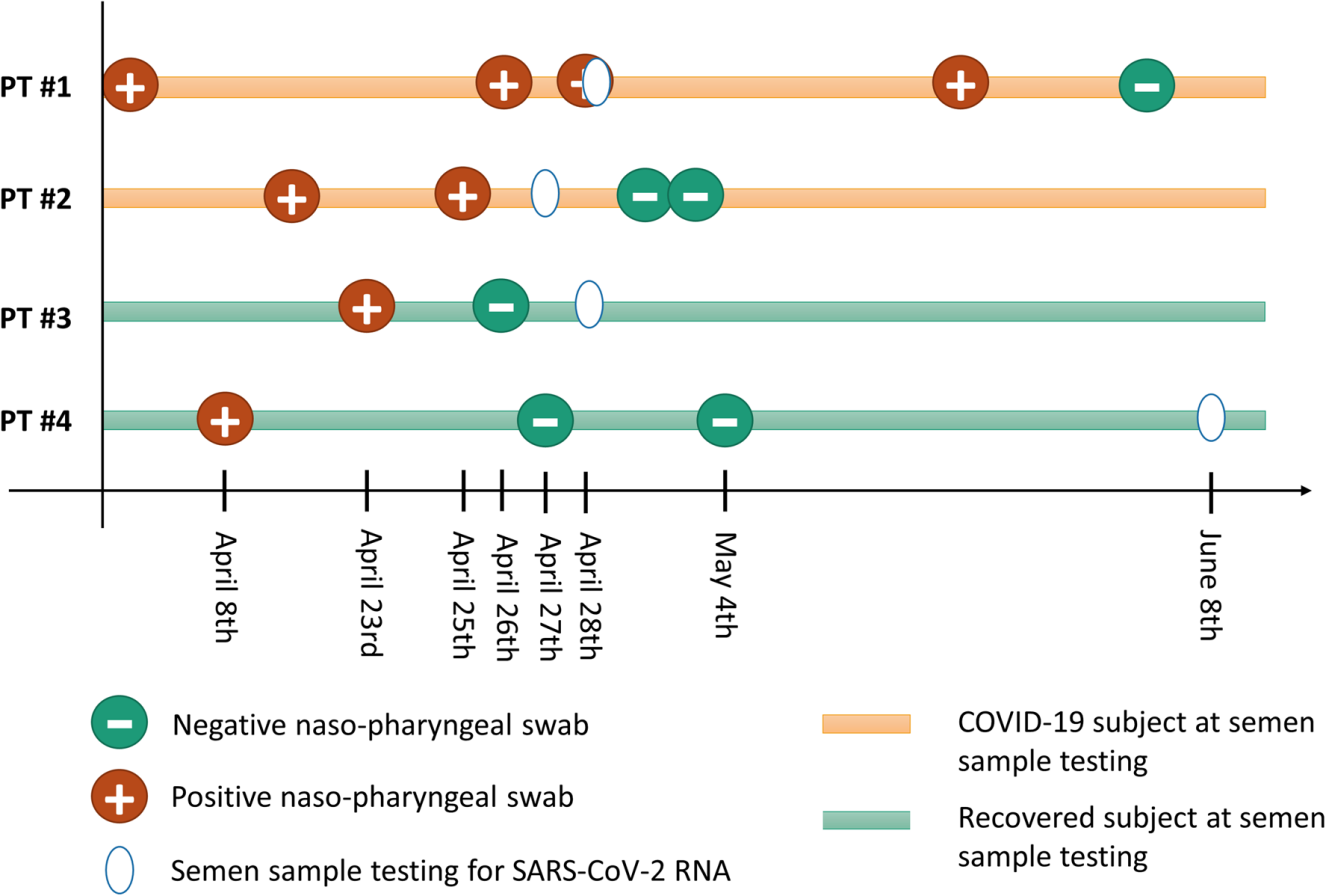
Patient’s information

Patient information is summarized in Fig. 3 and Table 1. The semen sample after a positive nasopharyngeal test was obtained from pt#1 on the same day of the positive nasopharyngeal swab was performed, and from pt #2 within 48 h of the last positive swab. All tested semen samples (both COVID-19 patients and controls) were negative for SARS-CoV-2 RNA, regardless of the nasopharyngeal swab result. To investigate the presence of the virus in different fractions of seminal fluid, we also extracted RNA from whole samples, seminal plasma and post-centrifugation pellets containing only the corpuscular part of the seminal fluid, namely spermatozoa, germ cells, leukocytes and epithelial cells. We did not detect the virus in any of these fractions. Internal control was detected in all samples. Semen parameters of all recruited subjects are shown in Table 2.

**Table 1:** Medical history of the recruited patients

Patient	Age (years)	Medical history	Andrological history	COVID-19 onset	Therapy
#1	58	Hodgkin’s lymphoma in 2004 and recurrence in 2015	No andrological diseases Two children before lymphoma	Fever on March 5th, 2020 Hospitalized on March 17th, 2020 after positive swab (no fever reported)	Tocilizumab (from March 22nd); Lopinavir/ritonavir and hydroxychloroquine (from March 18th to 23rd); other: Pantoprazole, Alprazolam, Zolpidem, Enoxaparin
#2	59	Recent femoral prosthesis implant	No andrological diseases Two children	Contact with a positive subject during rehabilitation. Onset of fever and cough on April 8th 2020. Hospitalization after positive swab on April 10th 2020	Hydroxychloroquine and Enoxaparin until April 20th
#3	61	History of ulcerative colitis, hemicolectomy and cerebrovascular disease	Left varicocele repair at age 20 Two children	Fever on March 8th, 2020 Hospitalized on March 18th, 2020 after positive swab (no fever reported at admission)	Darunavir/cobicistat (from March 19th to April 22nd); hydroxychloroquine (from March 19th to April 28th); Tocilizumab (from March 19th to April 25th); other: mesalazine, omeprazole, acetylsalicylic acid
#4	28	Multiple myeloma	No andrological diseases No children	Fever on April 5th, 2020. Due to mild symptoms, patient was assisted through a home care network. Mandatory self-quarantine ended after two consecutive negative swabs (April 27th and May 4th 2020)	Paracetamol as needed

**Table 2:** Sperm parameters of recruited subjects

Patient	Time from last positive swab to semen collection	Semen volume (ml)	Total sperm number (× 10^6^/ejaculate)	Progressive motility (%)		Non-progressive motility (%)	Abnormal forms (%)	Leukocytes (× 10^6^/ml)	Germ cells	Epithelial cells	Vitality (%)
				Linear	Non-linear						
#1	< 24 h	1.0	Azoospermia					0.4	–	–	–
#2	< 48 h	1.0	90	–	5	10	95	1.0	Yes	Yes	16%
#3	5 days	0.5	95	15	30	–	90	0.6	Yes	Yes	61%
#4	61 days	2.7	121.5	50	5	–	88	0.6	Yes	Yes	68%
CTR#1	N/A	4.0	180	45	10	–	88	0.7	Yes	–	85%
CTR#2	N/A	5.0	175	55	–	–	85	0.6	Yes	Yes	80%

### RNA extraction

To verify the efficiency of RNA extraction from sperm we used PRM1 and PRM2 mRNA as the control. Protamines 1 or 2 are the most abundant and specific nuclear proteins in human sperm [13]. PRM1 and PRM2 mRNA expression was found in all the semen samples (data not shown), demonstrating a good extraction capacity from this matrix.

### Control panel

The assays were evaluated against a panel using negative control samples and 0.5% saline solution. We tested diluted controls infected with a known titer of SARS-CoV-2 virus. This was required to assess if any substances in the seminal fluid might interfere with viral RNA extraction, inducing false negatives or false positives. Two known titer viral preparations from a panel were diluted 1:2 in seminal fluid (from SARS COV2-negative patients) and in 0.5% saline solution. All samples obtained by diluting viral preparation from the panel tested positive for SARS-CoV-2, with no RT-PCR inhibition detected.

The final virus concentrations were 2 × 10^4^ and 2 × 10^6^ copies/ml. Viral preparations diluted in saline and seminal fluid showed a similar Ct value to the initial viral concentration, as shown in Table 3.

**Table 3:** RT-PCR Ct values for S and E genes detected in control samples of whole seminal fluid and 0.5% saline solution infected with known titers of SARS-CoV-2

SPECIMEN	Ct S gene	Ct E gene
SARS-CoV-2 in seminal fluid (4 × 10^4^ copies/ml; diluted 1:2)	31.7	33.4
SARS-CoV-2 in saline (4 × 10^4^ copies/ml; diluted 1:2)	31.9	32.8
SARS-CoV-2 in seminal fluid (4 × 10^6^ copies/ml; diluted 1:2)	25.8	26.8
SARS-CoV-2 in saline (4 × 10^6^ copies/ml; diluted 1:2)	25.0	25.9

## Discussion

The outbreak of coronavirus disease (COVID-19) caused by SARS-CoV-2 has raised a number of concerns about public health, including sex-related mortality [14, 15]. Epidemiological studies suggested that males are more likely to test positive for COVID-19 [16]. This has prompted questions about the possible repercussions of SARS-CoV-2 for the male reproductive system. SARS-CoV-2 enters cells by means of a viral receptor, angiotensin-converting enzyme 2 (ACE2), which is highly expressed in a wide range of human tissues. In the testis, ACE2 expression has been found on seminiferous duct cells, spermatogonia, and Leydig and Sertoli cells, confirming the potential risks to the reproductive system associated with SARS-CoV-2 infection [17]. SARS-CoV-2 also requires transmembrane protease serine 2 (TMPRSS2) to enter cells. This proteolytic enzyme is involved in numerous physiological processes [18]. TMPRSS2 cleaves and modifies spike protein, enabling the permanent fusion of the virus and host cell [19]. It is highly expressed in the prostate epithelial cells, and its expression is regulated by androgens. The question thus arises: can SARS-CoV-2 reach the seminal fluid?

Several authors have investigated the presence of SARS-CoV-2 in semen [10, 20–32] (Table 4). They all conducted a search for viral RNA through RT-PCR, albeit screening for different genes. It must be stressed that of the 15 publications to date that have investigated this aspect, only 1 reported finding viral RNA in semen from both acute (26.7%) and recovering (8.7%) patients [10]. Furthermore, SARS-CoV-2 has currently only been investigated in semen in 31 acute COVID-19 cases and relatively few recovering subjects, including the aforementioned study. Overall, only 4 acute and 3 recovered patients have been reported to have seminal fluid positive for viral RNA over a total of 341 subjects evaluated (Table 4). Since most positive subjects came from the same study, the peculiar clinical conditions (disease severity) and methodological weaknesses of this paper have been discussed [11]. Recently, another paper reported a SARS-CoV-2-positive seminal fluid in a caseload of 15 mild-asymptomatic subjects, however, the presented data are scant [33]. However, further factors influencing the heterogeneity of these papers should also be recognized, including different ethnicities, slightly different definitions for acute cases, and huge differences in timing for the testing of recovering cases. On the hypothesis that the virus sheds into semen, all these factors could greatly affect both viral load and viral clearance in semen, and hence the chance of its detection. Consequently, although the presence of SARS-CoV-2 in semen cannot yet be completely excluded, the available data may be interpreted cautiously, but optimistically—especially given the absence of solid proof of its presence in the testes of non-severe COVID-19 cases [34].

**Table 4:** Summary of relevant literature evidence available on SARS-CoV-2 RNA detection in seminal fluid

References	Total Patients	Age (mean or range)	Acute (positive semen)	Acute (negative semen)	Recovered (positive semen)	Recovered (negative semen)	Method
Gacci et al. [20]	43	30–64			1	42	RT-PCR
Li et al. [21]	23	69.3			0	23	RT-PCR
Ruan et al. [22]	70	30.5			0	70	RT-PCR
Temiz et al. [26]	30	37.2			0	30	RT-PCR
Rawlings et al. [27]	6	38			0	6	RT-PCR
Pavone et al. [28]	9	42			0	9	RT-PCR
Kayaaslan et al. [29]	16	33.5	0	10	0	6	RT-PCR
Holtmann et al. [30]	20	42.2	0	2	0	18	RT-PCR
Ma et al. [23]	12	31.5	0	1	0	11	RT-PCR
Guo et al. [24]	23	41			0	23	RT-PCR
Pan et al. [25]	34	37			0	34	RT-PCR
Li et al. [10]	38	n/a	4	11	2	21	RT-PCR
Paoli et al. [32]	1	32			0	1	RT-PCR
Song et al. [31]	12	22–38	0	1	0	11	RT-PCR
Present study	4	51.5	0	2	0	2	RT-PCR
Total	341		4	27	3	307	

Gonzales et al. [35] reviewed literature data on the presence of SARS-CoV-2 in semen. They found a very low risk in seminal fluid, and a negligible risk in recovered men [35]. These results suggest that the likely absence of SARS-CoV-2 in seminal fluid may be influenced by biological or methodological factors. In relation to biological factors, we know that the testicles may be vulnerable to SARS-CoV-2 infection. However, given the concentration of SARS-CoV-2 receptors present in testicular tissue, why is the infection not clinically evident in the testes [36]? Studies based on single-cell RNA sequencing (sc RNAseq) in humans did not find any ACE2/TMPRSS2 co-expression in any type of testicular tissue [37]. In theory, viruses could reach semen from the blood, as the blood–testis barrier does not seem to constitute an insurmountable obstacle to viruses in the presence of systemic or local inflammation [38]. To date, few studies have investigated the presence of SARS-CoV-2 in blood. Bwire et al. reported a low (1.0%) detection of SARS‐CoV‐2 in blood samples [6]. It could be that the virus only spreads to blood under certain circumstances, such as the acute phase or severe disease, and then to other organs such as the testis [39].

Methodological factors are also important. While qRT-PCR assay, as discussed above, is the first-line screening method of choice for SARS-CoV-2 detection due to its high sensitivity and rapid detection [7], there is a real risk of false negative and false positive results [40]. False negative results may be due to sample inhibitors, poor amplification efficiency, and reduced precision in low concentration samples. False positives could arise from contaminants or poor test specificity [41]. In any case, the sensitivity and specificity of the RT-PCR methods used to detect SARS-CoV-2 in seminal fluid have not been evaluated [42].

In our study, we investigated the presence of SARS-COV-2 in seminal fluid from four COVID-19 patients: two mild cases with a positive recent nasopharyngeal swab and two whose last swab was negative. We did not find SARS-CoV-2 RNA in any of these samples. Semen analyses from the positive patients showed some abnormalities; specifically, patient #1 was azoospermic and patient #2 asthenozoospermic. It should be stressed that these semen characteristics are likely to be due to their medical history: patient #1 had undergone chemotherapy for lymphoma, while the asthenozoospermia of patient #2 was probably caused by his clinical condition, its treatment, and prolonged abstinence.

For a qualitative determination of the RT-PCR assays in semen we performed different attempts: (1) we verified the efficiency of RNA extraction from sperm and seminal plasma using PRM1 and PRM2 mRNA and a heterologous system, respectively, as control; (2) we tested samples obtained by diluting viral preparation from a panel tested positive for SARS-CoV-2, with no RT-PCR inhibition detected; (3) we investigated the presence of the virus in different fractions of seminal fluids, whole samples, seminal plasma and post-centrifugation pellets containing only the corpuscular part of the seminal fluid. We did not detect the virus in any of these fractions.

Our study not only demonstrated the absence of SARS COV2 in the seminal fluid of patients in the acute phase with a positive nasopharyngeal swab and in recovered patients with a negative swab, but for the first time confirmed the feasibility of this test for the molecular diagnosis of SARS-CoV-2 in seminal fluid. This result is important in two ways. First, it confirms the literature data on the absence of the virus in seminal fluid in patients with mild COVID-19, and second, it verifies the molecular method used through various tests. This information is important for reproductive medicine, especially in assisted reproductive technology and sperm cryopreservation.

The limitation of this method in relation to seminal fluid is that contamination could lead to a false positive. It should be stressed that semen collection is not sterile, and the sample could be contaminated with respiratory droplets or other body fluids from the patient, or by the patient’s hands. For this reason, any positive test result should be confirmed by repeating the test, alongside an evaluation of the patient’s symptoms and a thorough andrological history.

In our opinion, the molecular diagnosis of SARS COV2 in seminal fluid could be a useful tool for specialists in reproductive medicine to evaluate the safety of sperm.

## Data Availability

Not applicable.
